# Knowledge, Attitude and Practice of Artificial Intelligence Among Healthcare Professionals at a Tertiary Care Teaching Hospital in South Gujarat

**DOI:** 10.7759/cureus.73948

**Published:** 2024-11-18

**Authors:** Sajal Pandya, Chetna Patel, Brijesh Sojitra, Jaykumar Patel, Paras Shah, Akash Shah

**Affiliations:** 1 Pharmacology, Government Medical College Surat, Surat, IND; 2 Pharmacology, New Civil Hospital, Surat, IND; 3 Pharmacology and Therapeutics, Government Medical College Surat, Surat, IND

**Keywords:** ai applications in healthcare, ai ethical guidelines, artificial intelligence (ai), attitude, healthcare professionals (hcps), knowledge, practice (kap)

## Abstract

Background

Artificial intelligence (AI) is rapidly evolving within healthcare, promising improvements in patient care, diagnostic accuracy, and therapeutic interventions. As AI technology becomes more integrated into clinical workflows, understanding healthcare professionals’ (HCPs) knowledge, attitudes, and practices concerning AI is crucial, particularly in diverse healthcare environments like South Gujarat. This study evaluates HCPs' understanding, perception, and application of AI at a tertiary care teaching hospital in this region.

Methods

This observational, cross-sectional study utilized a non-validated, structured questionnaire based on the Knowledge, Attitude, and Practice (KAP) framework. A convenient sampling technique was employed to recruit 290 HCPs, including consultant doctors, medical faculty, residents, and interns. Data were collected electronically via Google Forms and analyzed using descriptive statistics.

Results

Most participants (176; 60.7%) were junior residents, with notable representation from departments like Pharmacology and Community Medicine. Regarding AI knowledge, 80 (27.6%) of participants reported full awareness, while 182 (62.8%) were partially aware. AI subtype knowledge varied, with 84 (28.9%) identifying "Self-awareness" and 50 (17.2%) "Limited Memory." Internet sources were the predominant information source for 171 (58.9%) of participants. Notably, 192 (66.2%) recognized AI's role in saving time and enhancing accuracy, although some expressed concerns about its lack of empathy and ethical implications.

Conclusions

The findings highlight substantial awareness but varying depths of understanding of AI among HCPs, who are interested in further AI education. Increased educational programs on AI’s ethical and practical aspects may enhance AI integration into clinical practice, aiding responsible adoption in healthcare settings.

## Introduction

Artificial intelligence (AI) has emerged as a key focus for scientists, with its convergence with various fields becoming increasingly prominent. Over the past decade, particularly following COVID-19, the integration of AI in medicine and medical education has accelerated, driven by the global shift to online learning and conferences. As AI technology evolves, so does the role of clinicians, medical postgraduates and medical faculties [1]. India’s healthcare sector struggles to achieve universal coverage and trails behind many developing and some least developed countries in health indicators such as infant mortality, under-five mortality, maternal mortality, life expectancy, malnutrition, sanitation and clean water access, chronic diseases, and vaccination coverage [2]. India's healthcare system faces significant challenges related to quality, accessibility, affordability, and inequity. While the country boasts some of the world's leading hospitals, spurring a burgeoning medical tourism industry, there is a critical shortage of qualified medical professionals. The doctor-to-population ratio in India is estimated at one doctor per 1,596 people, considering an 80% availability rate of doctors [3].

Integrating AI into healthcare can transform patient care and improve outcomes. AI-driven predictive analytics can significantly enhance the accuracy, efficiency, and cost-effectiveness of disease diagnosis and clinical testing. Furthermore, AI can support population health management and the development of clinical guidelines, delivering real-time, precise information and optimizing medication choices [4]. New technologies often require time to become integrated into healthcare, and despite AI’s promising potential, its adoption has been slower and more uneven than expected. Numerous visible and hidden barriers continue to impede its full incorporation into healthcare practices [5].

In the AI era, it is essential to understand healthcare professionals' perspectives on integrating AI into healthcare, as well as their knowledge, attitudes, and current practices regarding its use. This study was planned to assess how well consultants, medical faculties, resident doctors, and intern doctors at a tertiary care teaching hospital in South Gujarat understand AI. Additionally, this research seeks to evaluate their attitudes towards AI and its practical applications in healthcare. By identifying areas where knowledge may be lacking or where barriers to AI adoption exist, the findings of this study could help guide educational programs and inform policy decisions, ultimately enhancing the responsible use of AI in healthcare delivery.

## Materials and methods

Study design

This observational, and cross-sectional study utilized a questionnaire-based (self-administered) design to evaluate the knowledge, attitudes, and practices (KAP) of AI among healthcare professionals (HCPs) at a tertiary care teaching hospital in South Gujarat.

Inclusion criteria

HCPs who were willing to participate in the study. These included consultant doctors, medical faculties, resident doctors and intern doctors at the tertiary care teaching hospital in South Gujarat.

Exclusion criteria

Incomplete responses on the Google Forms questionnaire were excluded from the study.

Sampling

The sample size was calculated using the Raosoft software (Raosoft, Inc., Seattle, WA, USA; http://www.raosoft.com/samplesize.html). The required sample size was estimated at the 95% confidence level, with an estimated 50% prevalence and a margin of error of ±5%. The required minimum sample size was determined to be 290. A convenient sampling technique was employed to recruit participants.

Data collection

A non-validated and structured questionnaire was developed based on the reviewed relevant literature on the KAP framework related to AI in healthcare. The questionnaire was distributed to the HCPs electronically via Google Forms through email or WhatsApp (Meta Platforms, Inc., Menlo Park, CA, USA) ensuring ease of access and participation while minimizing the need for physical contact. KAP was assessed through a questionnaire completed by participants, which included targeted questions on their knowledge, attitude, and practice of AI.

Data analysis

The collected data were compiled and analyzed using Microsoft Excel version 2023 (Microsoft Corp., Redmond, WA, USA). Descriptive statistical methods were employed to summarize the data, including frequencies and percentages as applicable.

Timeline

Data collection occurred over one month (23/08/2024 to 22/09/2024), followed by a month (23/09/2024 to 22/10/2024) for data analysis and manuscript writing, making the total duration of the study two months.

Ethical considerations

The study was conducted after receiving approval from the Institutional Review Board (IRB) to ensure that all ethical standards were maintained (approval GMCS/STU/RRC-1/Approval/12381/24). Informed consent was obtained from all participants before the commencement of the questionnaire, and the study adhered to ethical guidelines to safeguard the rights and confidentiality of participants. Clinical Trials Registry of India (CTRI) registered number for the study is CTRI/2024/08/072741.

## Results

**Figure 1:**
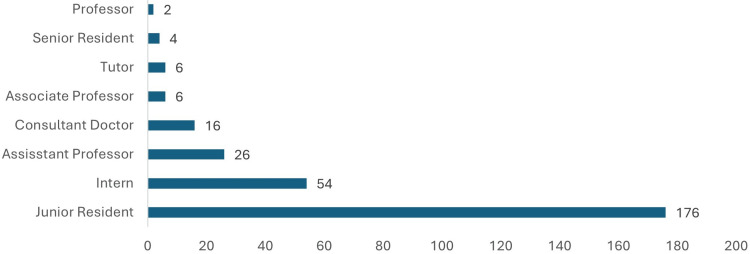
Designation of participants (Frequency) (n=290)

The study included 290 participants, as illustrated in Figure 1. The majority were Junior Residents (176, 60.7%), followed by Interns (54, 18.6%). Other designations included Assistant Professors (26, 9%), Consultant Doctors (16, 5.5%), and a smaller number of Associate Professors (six), Tutors (six), Senior Residents (four), and Professors (two). 

**Figure 2:**
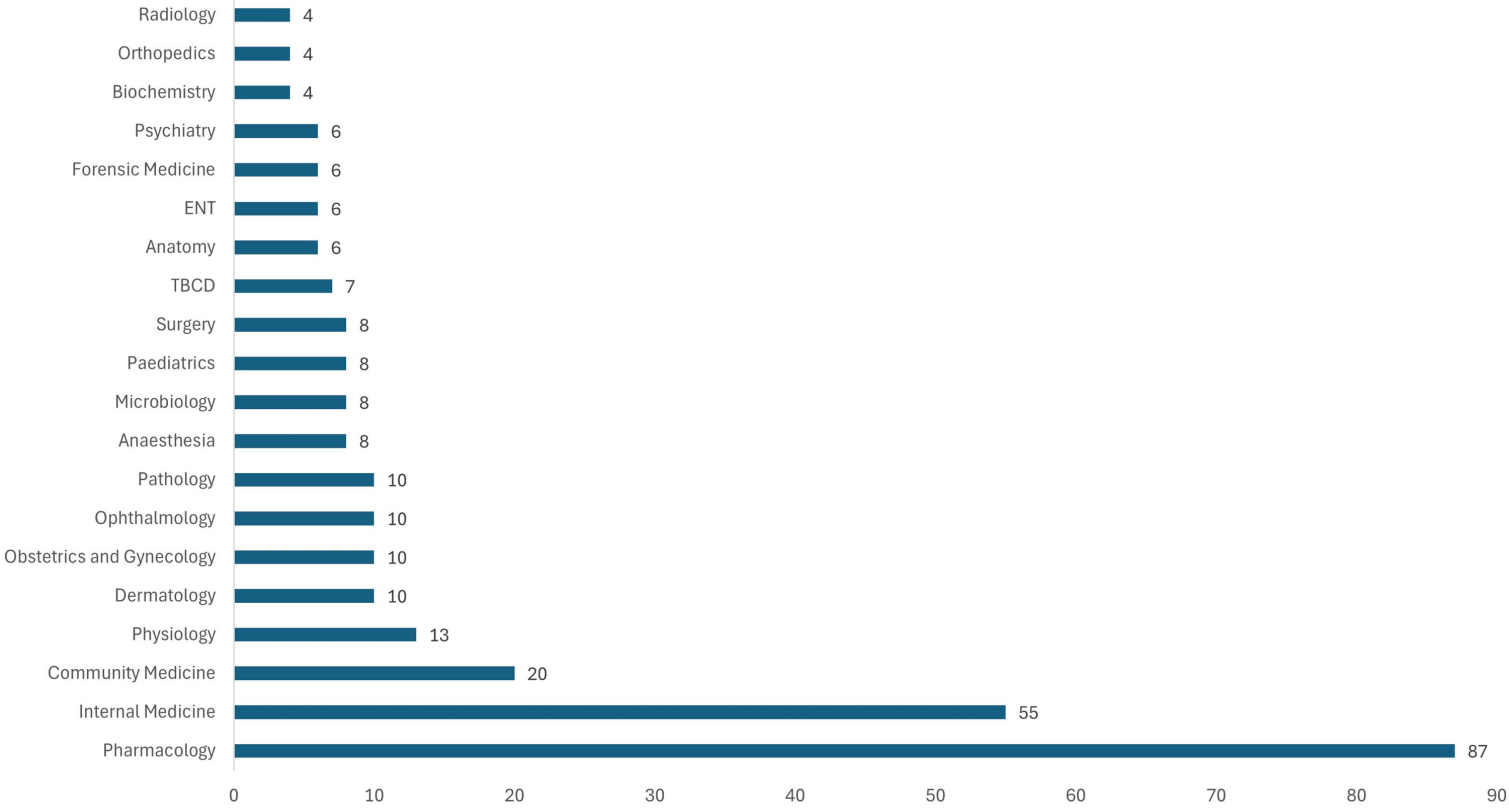
Department of participants (Frequency) (n=290)

Participants came from various medical departments, with the majority from Pharmacology (87, 30%). Other notable groups were Internal Medicine (55, 18.96%) and Community Medicine (20, 6.9%). Smaller representations included Physiology (13, 4.48%) and Dermatology, Obstetrics and Gynecology, Ophthalmology, and Pathology (10 each, 3.45%), as shown in Figure 2. 

**Figure 3:**
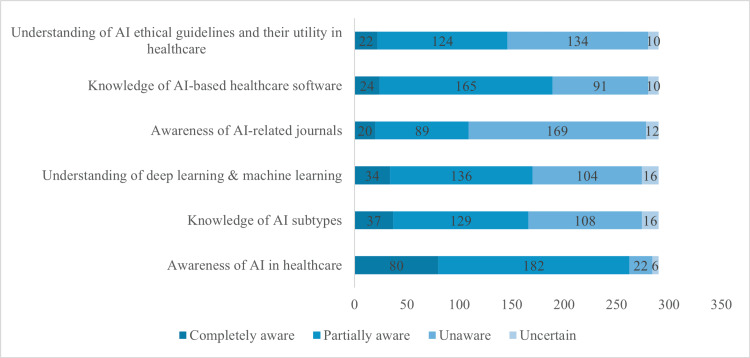
Awareness and Knowledge of AI and its Applications in Healthcare among Participants (n=290)

Figure 3 summarizes participants' awareness and knowledge of AI in healthcare (n=290). Self-reported awareness was reported by 80 participants (27.6%), while 182 (62.8%) were partially aware. A small group (22 participants, 7.6%) were unaware, and six (2.1%) were uncertain. Regarding AI subtypes, 37 participants (12.8%) had reported complete knowledge, and 129 (44.4%) showed partial knowledge, with 108 (37.2%) unaware. For deep learning and machine learning, 34 participants (11.7%) reported complete understanding, while 136 (46.9%) had partial understanding. In AI-related journals, 20 participants (6.9%) were reported completely aware, and 89 (30.7%) were partially aware, while 169 (58.3%) were unaware. For AI-based healthcare software, reported complete knowledge was noted by 24 participants (8.3%), with 165 (57.0%) partially knowledgeable. Understanding of AI ethical guidelines was reported complete for 22 participants (7.6%), while 124 (42.8%) demonstrated partial understanding.

Table 1 outlines AI subtype preferences among participants (n=290). The most selected option for preferred AI subtype was "Self-awareness" (84 participants, 28.97%), followed by "Limited Memory" (50 participants, 17.24%) and "Theory of Mind" (36 participants, 12.41%). "Reactive Machines" was chosen by 23 participants (7.93%). Additionally, 18 participants (6.2%) preferred multiple subtypes, while 79 participants (27.24%) did not opt.

**Table 1:** Participants' Preferred AI Subtype(s) Based on Survey Responses (n=290)

AI Subtype(s) Preferred by Participants (Answer options to the question: "Which AI subtype(s) do you prefer?")	Frequency (Percentage)
Limited memory	50 (17.24%)
Limited memory & Theory of mind	2 (0.69%)
Limited memory & Reactive machines	2 (0.69%)
Theory of mind	36 (12.41%)
Theory of mind & Self-awareness	10 (3.45%)
Theory of mind, Self-awareness & Reactive machines	2 (0.69%)
Theory of mind & Reactive machines	2 (0.69%)
Self-awareness	84 (28.97%)
Reactive machines	23 (7.93%)
None of the above	79 (27.24%)
Grand Total	290 (100.00%)

Table 2 shows sources of AI information among participants (n=290). The Internet was the main source, with 171 participants (58.97%) citing it. Other sources included "Workshop/Conference/CME" (40 participants, 13.79%) and "Family/Friends" (33 participants, 11.38%). While 36 participants (12.41%) indicated mixed sources.

**Table 2:** Sources of Information About AI Among Participants (n=290)

Source of Information About AI (Answer options to the question: "Where did you get your information about AI?")	Frequency (Percentage)
Family / friends	33 (11.38%)
Family / friends, Newspaper & Internet	4 (1.38%)
Family / friends, Newspaper, Internet & Workshop/Conference/Continuing Medical Education (CME)	4 (1.38%)
Family / friends & Internet	11 (3.79%)
Newspaper	2 (0.69%)
Newspaper & Internet	4 (1.38%)
Newspaper, Internet & Workshop/Conference/Continuing Medical Education (CME)	2 (0.69%)
Internet	171 (58.97%)
Internet & Workshop/Conference/Continuing Medical Education (CME)	7 (2.41%)
Internet, Workshop/Conference/Continuing Medical Education (CME) & Other	2 (0.69%)
Workshop/Conference/Continuing Medical Education (CME)	40 (13.79%)
Workshop/Conference/Continuing Medical Education (CME) & Other	2 (0.69%)
Other	8 (2.76%)
Grand Total	290 (100.00%)

Table 3 shows participants' knowledge of ChatGPT features (n=290). Most participants, 225 (77.59%), selected "All of the above," identifying it as a chat generative pretrained transformer. Additionally, 23 (7.93%) recognized the latest version as GPT-4. Meanwhile, 18 (6.21%) acknowledged its generative capabilities, and 10 (3.45%) identified it as a large language model. Finally, 12 (4.14%) showed mixed familiarity with its features.

**Table 3:** Participant Knowledge of ChatGPT Features and Capabilities (n=290)

Statement/Fact About ChatGPT (Answer options to the question: "Which of the following statements/facts about ChatGPT do you agree with?")	Frequency (Percentage)
ChatGPT is a chat generative pretrained transformer	18 (6.21%)
ChatGPT is a chat generative pretrained transformer & It is a large language model tool	4 (1.38%)
ChatGPT is a chat generative pretrained transformer, It is a large language model tool, Latest version of ChatGPT is GPT-4 & All of the above	2 (0.69%)
ChatGPT is a chat generative pretrained transformer & Latest version of ChatGPT is GPT-4	2 (0.69%)
It is a large language model tool	10 (3.45%)
It is a large language model tool & Latest version of ChatGPT is GPT-4	6 (2.07%)
Latest version of ChatGPT is GPT-4	23 (7.93%)
All of the above	225 (77.59%)
Grand Total	290 (100.00%)

Table 4 shows participants' awareness of BharatGPT Hanooman features (n=290). A significant 210 participants (72.41%) recognized "All of the above," demonstrating a strong understanding of the model. Specifically, 39 participants (13.45%) noted its multimodal capabilities, while 12 (4.14%) mentioned training in 22 Indian languages. Additionally, eight participants (2.76%) identified its development by Reliance in collaboration with nine Indian Institutes of Technology (IITs), and 14 participants (4.82%) provided combined responses, indicating varying awareness levels.

**Table 4:** Participant Awareness of BharatGPT Hanooman Features and Development (n=290) IITs: Indian Institutes of Technology, LLM: Large language model

Statement About BharatGPT Hanooman (Answer options to the question: "Which of the following statements about BharatGPT Hanooman do you agree with?")	Frequency (Percentage)
It is developed by Reliance and nine IITs across the country	8 (2.76%)
It is developed by Reliance and nine IITs across the country & It is trained on 22 Indian languages	2 (0.69%)
It is developed by Reliance and nine IITs across the country, It is trained on 22 Indian languages, Hanooman has multimodal AI capabilities for generating text-to-text, text-to-speech, text-to-video and vice versa content, One of the major challenges for building Indian LLMs is sourcing quality datasets in Indian languages & All of the above	2 (0.69%)
It is developed by Reliance and nine IITs across the country, Hanooman has multimodal AI capabilities for generating text-to-text, text-to-speech, text-to-video and vice versa content & One of the major challenges for building Indian LLMs is sourcing quality datasets in Indian languages	2 (0.69%)
It is trained on 22 Indian languages	12 (4.14%)
It is trained on 22 Indian languages, Hanooman has multimodal AI capabilities for generating text-to-text, text-to-speech, text-to-video and vice versa content & One of the major challenges for building Indian LLMs is sourcing quality datasets in Indian languages	2 (0.69%)
Hanooman has multimodal AI capabilities for generating text-to-text, text-to-speech, text-to-video and vice versa content	39 (13.45%)
Hanooman has multimodal AI capabilities for generating text-to-text, text-to-speech, text-to-video and vice versa content & One of the major challenges for building Indian LLMs is sourcing quality datasets in Indian languages	2 (0.69%)
Hanooman has multimodal AI capabilities for generating text-to-text, text-to-speech, text-to-video and vice versa content & All of the above	4 (1.38%)
One of the major challenges for building Indian LLMs is sourcing quality datasets in Indian languages	7 (2.41%)
All of the above	210 (72.41%)
Grand Total	290 (100.00%)

**Figure 4:**
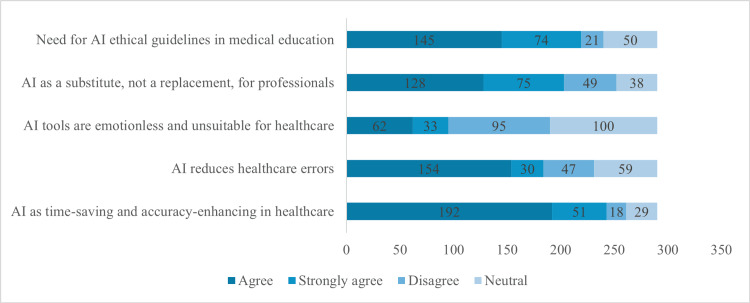
Participant Perspectives on AI Utility, Suitability, and Ethical Guidelines in Healthcare (n=290)

Figure 4 shows participants' perspectives on AI in healthcare (n=290). A majority, 192 participants (66.2%), believe AI saves time and enhances accuracy, while 154 (53.1%) think it reduces healthcare errors. Conversely, 62 participants (21.4%) view AI as emotionless and unsuitable, with 95 (32.8%) disagreeing. Regarding AI's role, 128 participants (44.1%) see it as a substitute for professionals, and on the need for ethical guidelines, 145 (50%) agree that these should be included in medical education.

**Figure 5:**
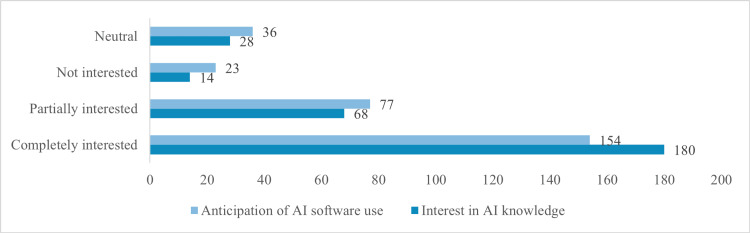
Interest in AI Knowledge and Aspiration of AI Software Use Among Participants (n=290)

Figure 5 shows participants' interest in AI knowledge and aspiration of using AI software (n=290). A significant 180 participants (62.1%) are fully interested in learning about AI, while 68 (23.4%) are partially interested. Only 14 (4.8%) are not interested, and 28 (9.7%) remain neutral. Regarding AI software usage, 154 participants (53.1%) fully aspire to use, with 77 (26.6%) showing partial interest. Meanwhile, 23 (7.9%) are not interested, and 36 (12.4%) have a neutral stance.

**Figure 6:**
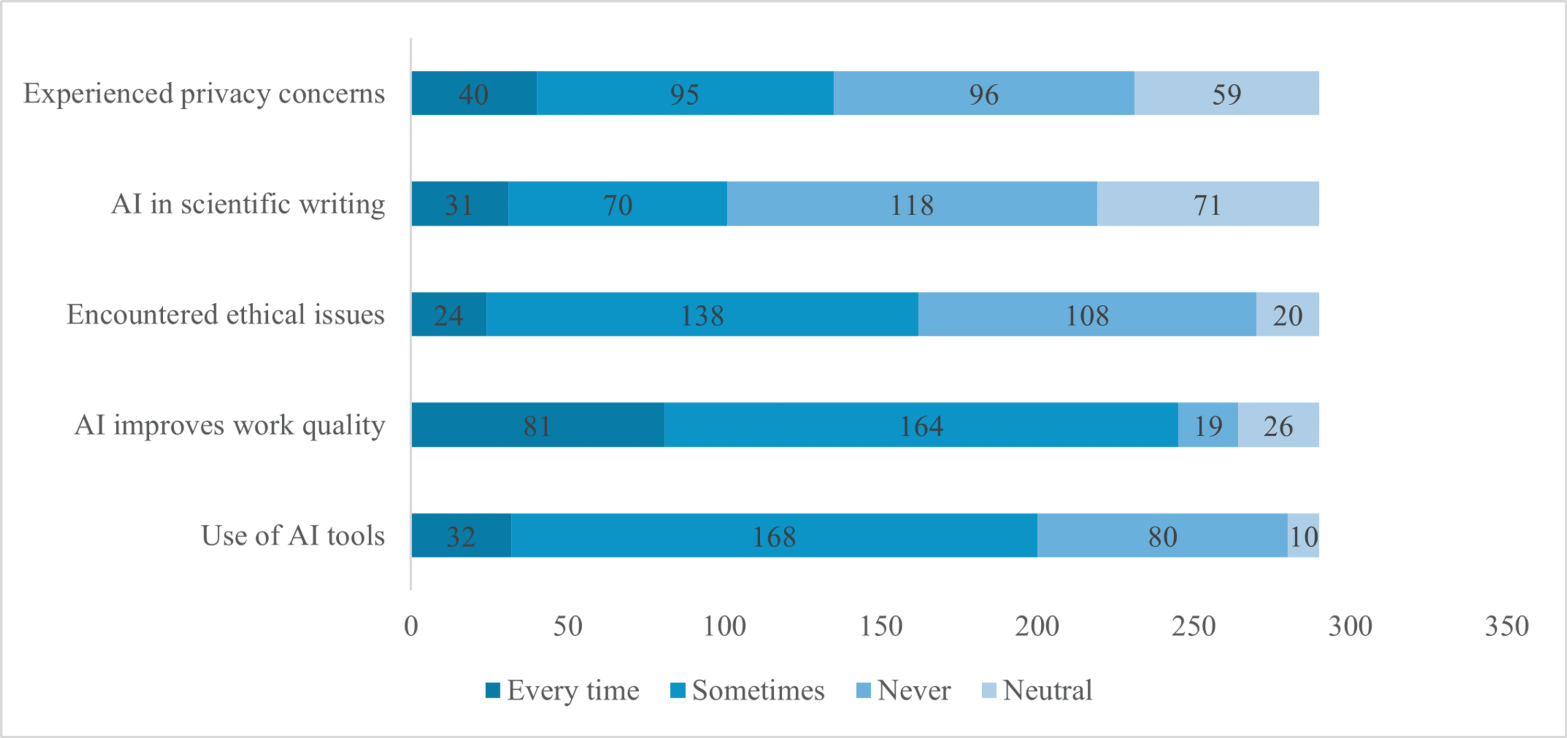
Frequency of AI Tool Usage and Related Perceptions Among Participants (n=290)

Figure 6 illustrates AI tool usage and perceptions among participants (n=290). Most participants, 168 (57.9%), use AI tools sometimes, while 32 (11%) use them regularly, and 80 (27.6%) never do. Regarding benefits, 81 participants (27.9%) feel AI improves work quality, and 164 (56.6%) think it sometimes enhances their work. Ethical issues have been encountered by 24 participants (8.3%), while 138 (47.6%) have not faced such challenges. In scientific writing, 31 participants (10.7%) use AI consistently, while 118 (40.7%) do not use it at all. Privacy concerns affect 40 participants (13.8%), with 95 (32.8%) occasionally expressing worries.

Table 5 shows the usage frequency of various language model tools among participants (n=290). ChatGPT is the most popular choice, used by 195 participants (67.24%), 14 participants (4.83%) use Google Bard, while smaller groups use combinations involving Bing AI and Copilot. Additionally, 40 participants (13.79%) reported using multiple tools amongst these.

**Table 5:** Usage Frequency of Various Language Model Tools Among Participants (n=290)

Language Model Tool(s) Used (Answer options to the question: "Which language model tool(s) have you used?")	Frequency (Percentage)
ChatGPT	195 (67.24%)
ChatGPT & Google Bard	16 (5.52%)
ChatGPT, Google Bard & Bing AI	4 (1.38%)
ChatGPT, Google Bard, Bing AI & Copilot	4 (1.38%)
ChatGP, Google Bard, Bing AI, Copilot & Other	2 (0.69%)
ChatGPT, Google Bard & Copilot	2 (0.69%)
ChatGPT & Bing AI	2 (0.69%)
ChatGPT & Copilot	4 (1.38%)
ChatGPT, Copilot & Other	2 (0.69%)
ChatGPT & Other	4 (1.38%)
Google Bard	14 (4.83%)
Bing AI	2 (0.69%)
Copilot	4 (1.38%)
Other	35 (12.07%)
Grand Total	290 (100.00%)

In response to the question about AI serving as a substitute, rather than a replacement, for physicians and academicians in the healthcare system, 44.14% of participants agreed.

Among the 290 respondents, 87 people (30%) had attended or conducted a workshop or Continuing Medical Education (CME) session related to AI. In contrast, 203 participants (70%) reported that they had not participated in any such activities.

## Discussion

The findings from this study offer critical insights into how healthcare professionals at a tertiary care hospital perceive and utilize AI in their practice. With input from 290 participants, the study highlights varied levels of awareness, familiarity with AI subtypes, preferences for AI tools, and ethical concerns surrounding AI's integration into healthcare.

Awareness and knowledge of AI in healthcare

A significant portion of participants (62.8%) demonstrated partial awareness of AI, while 27.6% claimed full awareness. This suggests that although AI is becoming more prevalent in healthcare, medical professionals still need a more comprehensive understanding. The transformative potential of AI has been noted, but the limited awareness of its subtypes in this study highlights the need for targeted AI education in medical curricula as there remains a sizable group lacking complete knowledge, especially in understanding AI subtypes like deep learning and machine learning, underscoring the need for better clarity [6,7].

AI implementation in healthcare requires extensive physician training, adherence to established practice ethics and guidelines, payment regulations for public and private organizations, and continuous updates over time. Organizing training programs, seminars, and webinars on AI, machine learning (ML), and deep learning (DL) is essential to equip healthcare professionals with the necessary skills and knowledge. To address key deficiencies and improve AI training in medical schools-countering any misconceptions, a foundational step would be to integrate electronic health records (EHR) training into the curriculum. Additionally, AI education could be expanded through CME programs. Ultimately, the primary challenge in adopting AI in healthcare is not the technology’s capability but ensuring its routine use in clinical practice [8].

Use of AI tools

ChatGPT is a valuable tool in daily clinical practice, offering insights for healthcare applications such as drug discovery, disease diagnosis, radiological imaging analysis, personalized medicine, and patient monitoring. It enhances diagnostic accuracy and treatment efficacy through its capabilities in translation, transcription, and summarization of feedback. ChatGPT and other OpenAI tools significantly improve healthcare delivery, especially in resource-limited settings, by providing providers and patients information on drug-drug interactions (DDIs) [9].

The study shows that AI tools are gaining traction, with 57.9% of participants reporting occasional use, and ChatGPT being the most popular tool, used by 67.24% of respondents. This aligns with current trends where healthcare professionals leverage AI tools for scientific writing and clinical decision-making tasks [10]. In contrast to our study, only 53 individuals (11.3) had practical experience with AI, all of whom agreed that it facilitated their tasks. Meanwhile, the remaining 417 participants (88.7%), had never used AI in any capacity [11]. A total of 192 participants (66.2%) agree that AI improves efficiency and boosts precision. AI benefits extend beyond patient care, playing a crucial role in reducing physician burnout by automating repetitive tasks and streamlining workflows. This boosts healthcare efficiency, lowers costs, and allows doctors to focus on direct patient care. AI can also handle administrative tasks, expedite document searches, and serve as a real-time medical scribe, enhancing productivity across healthcare settings [12].

In our study, 44.14% of participants viewed AI as a supplement, not a replacement, for physicians and academicians in healthcare. Physicians rely on information that AI can process but still require judgment based on experience, considering each patient's unique needs, values, and socioeconomic factors. Many clinical symptoms, like pain and fatigue, are subjective and hard to quantify. Additionally, clinical signs and lab results can vary significantly within the same patient [13]. While AI is unlikely to replace physicians soon, medical professionals need to understand AI fundamentals and apply AI-driven tools to enhance patient outcomes. Ultimately, physicians who utilize AI effectively may surpass those who do not [14].

Ethical concerns and challenges

Ethical considerations are an area of concern, with 8.3% of respondents reporting issues related to AI in healthcare. Only 7.6% of participants were familiar with AI ethical frameworks, reinforcing the call for greater emphasis on ethics in AI education.

The rapid growth of AI in clinical and biomedical fields offers valuable support for healthcare professionals. However, alongside its potential, AI brings new ethical challenges, particularly in areas like access to data, infrastructure, privacy, data protection, patient autonomy, informed consent, equity, and the human elements of empathy and compassion. Patients are unlikely to favor “machine-human” interactions over “human-human” care, which may limit AI’s acceptance. Addressing this requires a strong focus on ethical and humane principles to balance AI’s benefits with essential patient-centered values [15,16]. Human values influence AI at every stage, and models can reflect biases or outdated utilities. It is essential to ensure AI models align with patient values and goals, reflecting our shared responsibility in healthcare [17]. Blockchain technology enhances data security, privacy, and interoperability. Its decentralized structure reduces risks of breaches, cyberattacks, and fraud, while its immutability ensures data integrity. Blockchain’s transparent, traceable records are essential for authenticating healthcare data and fostering patient trust, compliance, and engagement. Exploring its integration with other emerging technologies could further highlight practical benefits [18].

AI in medical education

The study revealed a strong interest in AI education, with 62.1% of respondents expressing a desire to learn more about AI. AI is currently applied across education, enhancing exam integrity, online discussions, research, student performance analysis, campus connectivity, lecture transcription, and personalized learning. It enables universal access, detects plagiarism, supports scientific writing, and offers timely, actionable feedback to students [9]. Massive Online Open Courses (MOOCs) offer accessible, context-focused medical education, enabling open dialogue between educators and learners and providing reliable information anytime, anywhere through trusted institutions [19]. AI advancements are set to transform publishing by streamlining and enhancing the peer-review process, improving review quality, and enabling innovative publication methods [20].

AI and healthcare efficiency

Most participants (66.2%) agreed that AI enhances healthcare accuracy and efficiency, aligning with studies that demonstrate AI’s role in improving diagnostic precision and minimizing human error. However, 21.4% expressed concerns that AI might dehumanize healthcare, viewing it as emotionless and unsuitable for patient care. There’s a concern that if AI becomes too central in care, it might, depending on how it’s used, gradually weaken the personal connection between physicians and patients [15].

The future of healthcare robotics focuses on remote presence and task automation, such as clinical disinfection and virtual ward rounds. This flexibility enables consistent, adaptable services, allowing care to continue uninterrupted, even during disruptions like pandemics [21]. Though still in its early stages, AI and robotics in healthcare have promising potential. Key areas for rapid adoption include elderly care, drug discovery, clinical trials, digital consultations, remote monitoring, nanotech research, and epidemic prediction [2].

Previous recommendations emphasize that AI developers should establish their ethical guidelines, monitored by peers or regulators. Certifications could reward adherence, encouraging developers to showcase their ethical practices, while regulators could highlight these efforts within a structured monitoring framework. Collaboration with local communities and across public and private sectors including healthcare providers, researchers, and tech firms-is crucial to developing accessible and effective AI solutions. Conducting health equity impact assessments can also help identify and address any disparities AI technologies may cause among different populations [22].

Limitations

The cross-sectional design limits causal inferences, and the sample, largely young doctors and pre-clinical department staff, may not reflect the broader clinical population. Additionally, the use of a non-validated questionnaire restricts the reliability of the findings. Therefore, the generalizability of the results may be varied to other settings.

## Conclusions

This study highlights a clear need to enhance AI education in healthcare. By focusing on practical applications and openly addressing ethical questions, healthcare professionals can be better prepared to integrate AI responsibly into clinical practice. Expanding AI training within healthcare education will help bridge knowledge gaps and encourage a thoughtful approach to using AI in patient care.

## Appendices

Appendix:  Questionnaire

1)     Demographic Details:

a)     Name

b)     Age

c)     Gender

d)     Designation

e)     Department

2)     Knowledge Based questions

a)            Are you aware about use of artificial intelligence in healthcare system?

i.             Completely aware

ii.            Partially aware

iii.           Unaware

iv.           Uncertain

b)            Do you know about basic subtypes of AI?

i.             Completely aware

ii.            Partially aware

iii.           Unaware

iv.           Uncertain

c)            Which of the following is not a subtype of AI?

i.             Limited memory

ii.            Theory of mind

iii.           Self-awareness

iv.           Reactive machines

v.            None of the above

d)            Do you know about the deep learning and machine learning?

i.             Completely aware

ii.            Partially aware

iii.           Unaware

iv.           Uncertain

e)            From where did you came to know about AI? (Source of information about AI)

i.             Family / friends

ii.            Newspaper

iii.           Internet

iv.           Workshop/ conference/ CME

v.            Other

f)            Are you aware of AI related journals?

i.             Completely aware

ii.            Partially aware

iii.           Unaware

iv.           Uncertain

g)            Do you know about AI based software in healthcare system?

i.             Completely aware

ii.            Partially aware

iii.           Unaware

iv.           Uncertain

h)            Which of the following is/ are true about ChatGPT?

i.             ChatGPT is chat generative pretrained transformer

ii.            It is a large language model tool

iii.           Latest version of ChatGPT is GPT-4

iv.           All of the above

i)             Do you know about ethical guidelines concerning the utility of AI in healthcare system?

i.             Completely aware

ii.            Partially aware

iii.           Unaware

iv.           Uncertain

j)             Which of the following is/are true about upcoming BharatGPT Hanooman?

i.             It is developed by Reliance and 9 IITs across the country

ii.            It is trained on 22 Indian languages

iii.           Hanooman has multimodal AI capabilities for generating text-to-text, text-to-speech, text-to-video and vice versa content

iv.           One of the major challenges for building Indian LLMs is the sourcing of quality datasets in Indian languages

v.            All of the above

3)     Attitude Based questions

a)            AI tools are time saving and are essential in Health care system to increase accuracy.

i.             Agree

ii.            Strongly agree

iii.           Disagree

iv.           Neutral

b)            AI tools reduces errors in healthcare system.

i.             Agree

ii.            Strongly agree

iii.           Disagree

iv.           Neutral

c)            AI tools are emotionless and not good to implement in health care system.

i.             Agree

ii.            Strongly agree

iii.           Disagree

iv.           Neutral

d)            AI is a substitute of physician / academician in health care system not a replacement.

i.             Agree

ii.            Strongly agree

iii.           Disagree

iv.           Neutral

e)            Incorporating ethical guidelines concerning the utility of AI into the postgraduate curriculum for medical education is essential.

i.             Agree

ii.            Strongly agree

iii.           Disagree

iv.           Neutral

f)            Would you be interested in gaining further knowledge about AI?

i.             Completely interested

ii.            Partially interested

iii.           Not interested

iv.           Neutral

g)            Do you anticipate utilizing AI software in the future?

i.             Completely interested

ii.            Partially interested

iii.           Not interested

iv.           Neutral

4)     Practice Based questions

a)            Have you ever used chatboats/ robotics/ other generative AI software?

i.             Every time

ii.            Sometimes

iii.           Never

iv.           Neutral

b)            Do you feel that AI makes your work easy and qualitative?

i.             Every time

ii.            Sometimes

iii.           Never

iv.           Neutral

c)            Have you ever used AI software in scientific writing?

i.             Every time

ii.            Sometimes

iii.           Never

iv.           Neutral

d)            Have you ever encountered ethical issues while using AI?

i.             Every time

ii.            Sometimes

iii.           Never

iv.           Neutral

e)            Have you ever experienced privacy concerns while using AI?

i.             Every time

ii.            Sometimes

iii.           Never

iv.           Neutral

f)            Which language model tool do you use?

i.             ChatGPT

ii.            Google Bard

iii.           Bing AI

iv.           Copilot

v.            Other

g)            Have you ever attended or conducted workshop/ CME regarding AI?

i.             Yes

ii.            No
